# Nonassociative learning as gated neural integrator and differentiator in stimulus-response pathways

**DOI:** 10.1186/1744-9081-2-29

**Published:** 2006-08-08

**Authors:** Chi-Sang Poon, Daniel L Young

**Affiliations:** 1 Harvard-MIT Division of Health Sciences and Technology, Massachusetts Institute of Technology, Cambridge, Massachusetts 02139, USA; 2 Entelos, Inc., 110 Marsh Drive, Foster City, CA 94404, USA

## Abstract

Nonassociative learning is a basic neuroadaptive behavior exhibited across animal phyla and sensory modalities but its role in brain intelligence is unclear. Current literature on habituation and sensitization, the classic "dual process" of nonassociative learning, gives highly incongruous accounts between varying experimental paradigms. Here we propose a general theory of nonassociative learning featuring four base modes: habituation/primary sensitization in primary stimulus-response pathways, and desensitization/secondary sensitization in secondary stimulus-response pathways. Primary and secondary modes of nonassociative learning are distinguished by corresponding activity-dependent recall, or nonassociative gating, of neurotransmission memory. From the perspective of brain computation, nonassociative learning is a form of integral-differential calculus whereas nonassociative gating is a form of Boolean logic operator – both dynamically transforming the stimulus-response relationship. From the perspective of sensory integration, nonassociative gating provides temporal filtering whereas nonassociative learning affords low-pass, high-pass or band-pass/band-stop frequency filtering – effectively creating an intelligent sensory firewall that screens all stimuli for attention and resultant internal model adaptation and reaction. This unified framework ties together many salient characteristics of nonassociative learning and nonassociative gating and suggests a common kernel that correlates with a wide variety of sensorimotor integration behaviors such as central resetting and self-organization of sensory inputs, fail-safe sensorimotor compensation, integral-differential and gated modulation of sensorimotor feedbacks, alarm reaction, novelty detection and selective attention, as well as a variety of mental and neurological disorders such as sensorimotor instability, attention deficit hyperactivity, sensory defensiveness, autism, nonassociative fear and anxiety, schizophrenia, addiction and craving, pain sensitization and phantom sensations, etc.

## 1. Background

Brain calculus – or integral-differential neural dynamics – is an emerging paradigm in computational neuroscience [1,2]. In behavioral neuroscience, the dynamics of sensorimotor integration are often ascribed to learning and memory. We hereby propose a general framework of nonassociative learning and nonassociative gating that demonstrate brain calculus and Boolean logic computations. The resultant neural network theory proves to illuminate a variety of behavioral and brain functions and disorders.

This article is written with a broad readership in mind. Beginning with a thorough review of the oft-conflicting literature on habituation and sensitization, the so-called "dual process" of nonassociative learning, Section 2 develops a unified framework of *primary *and *secondary sensitization *in analogy to pain sensitization. In Section 3, we introduce the notion of *response desensitization *[3] and show that this novel nonassociative learning mechanism provides a common kernel which may explain a variety of sensory remapping phenomena. Section 4 presents a novel behavioral paradigm called *nonassociative gating *which affords activity-dependent temporal filtering or Boolean logic-gating of the stimulus-response relationship. These emergent concepts cumulate in a general theoretical framework elaborated in Section 5, which expounds the computational roles of nonassociative learning as gated neural integrator and differentiator (low-pass and high-pass filter) in neural pathways. Section 6 discusses the functional roles of the various modes of nonassociative learning in brain intelligence as the building blocks of a "sensory firewall" for Cartesian mind-body internal model adaptation. Section 7 concludes the discourse.

## 2. Dual-process theory revisited

Although a universally agreed model of nonassociative learning is presently lacking (for reviews see [4-8]), a useful starting point is the classic *dual-process theory *of response habituation and sensitization [9-11]. In the following, we present a unified framework that extends and reconciles the dual-process theory and other models of nonassociative learning.

### 2.1. Habituation and sensitization: the 'dual-process theory'

#### 2.1.1. Dual-process theory

According to this classic theory, an animal's behavioral response to a repetitive stimulus may wane or wax through two complementary learning processes called *habituation *and *sensitization*. At the system level these processes are thought to correspond, respectively, to short-term depression (STD) of neurotransmission in a primary stimulus-response pathway and short-term potentiation (STP) or facilitation of neurotransmission in a secondary, collateral pathway or "state system" that presumably determines the animal's general level of excitation, arousal or motivation to respond. Here, "short-term" plasticity (potentiation or depression) refers to the short-term modifiability and short-term memory commonly seen in nonassociative learning although long-term memory (> 24 hr) is also possible [12-16]. In some model systems habituation is induced by an innocuous stimulus (such as gentle touch) and sensitization is induced by noxious stimulus (forceful touch or electrical shock). In other systems habituation and sensitization could be induced by the same stimulus (e.g., startle response to repetitive loud noise).

Certain conjectures of the dual-process theory have subsequently been verified in a variety of invertebrate and mammalian brain systems [17-30]. Circumstantial evidence for dual-process learning could also be inferred, albeit unwittingly, from other animal models of nonassociative learning reported in the literature (reviewed in [31]).

#### 2.1.2. Properties of response habituation

Typically, habituation may be induced by a stimulus that is presented continuously or intermittently with a variable interstimulus interval (ISI). The dual-process theory defined habituation by the following stimulus-response criteria [9,10,32]: 1) exponential development with repeated stimulus applications, causing exponential decrease of response to the stimulus; 2) spontaneous recovery with a short-term memory upon cessation of stimulus; 3) successive potentiation or accumulation with repeated training sessions; 4) dependence on stimulus frequency with rate and magnitude of habituation being directly related to frequency of stimulus bouts (and inversely related to ISI); 5) dependence on stimulus intensity with rate and magnitude of habituation being inversely related to stimulus intensity; 6) dependence on stimulus quantity with spontaneous recovery of habituation becoming much slower after an excessive number of stimulus bouts; 7) cross-modal generalization or transfer of habituation to other stimuli that share common habituating elements with the primary stimulus; 8) dishabituation or trumping of habituation by a novel stimulus; and 9) habituation of dishabituation upon repeated applications of the dishabituating stimulus.

These postulated properties of habituation have been borne out for the most part in many animal models from nematodes [33] to mammals – down to the level of a monosynaptic junction in the hippocampus [34]. It is generally assumed that response habituation is mediated primarily by homosynaptic depression in the stimulus-response pathway [32] although other mechanisms such as increased inhibition (see [5,35]) or decreased neuronal excitability [36] are also possible.

### 2.2. A unified framework for response sensitization

Although the above characterizations of habituation appear to prevail across animal phyla and sensory modalities, those of response sensitization are less clear. The lack of a consistent taxonomy for sensitization has made it difficult to decipher and relate the vast amounts of pertinent (and oft-conflicting) data from diverse animal models, sparking considerable confusion and controversy in the literature. Here we review two conventional characterizations, *intrinsic *and *extrinsic sensitization*, which have occasioned renewed interests (reviewed in [31]). We then propose a unified framework that reconciles the discrepancies between these characterizations of sensitization and the dual-process theory.

#### 2.2.1. Ambiguities of intrinsic and extrinsic sensitization

According to the dual-process theory, sensitization may be induced by repeated applications of a primary, or intrinsic, stimulus. Such "intrinsic sensitization" has been implicated in the increment phase of the rat acoustic startle response [37] and the monosynaptic ventral root reflex of the frog spinal cord [38]. It may also account for certain forms of sensitization such as the reported incremental sensitization of defensive striking in larval *Manduca Sexta *[39], iterative enhancement of the sea slug *Tritonia *swim response [40,41], warm-up phase in the local bending reflex of the medicinal leech *Hirudo medicinalis *[42], the "windup" or central sensitization of mammalian pain pathways [43-46] and the progressive intensification of the evoked irritant sensation upon repeated applications of the pungent chemical capsaicin to the tongue [47].

More commonly, sensitization is characterized as an increase in the response to a primary stimulus after priming by an extrinsic, often strong and noxious stimulus. This form of sensitization has been variously referred to as "conventional" or "nociceptive" sensitization [39] or "extrinsic" sensitization [10,27,37,42,48,49]. Its underlying mechanisms (as demonstrated in the *Aplysia *gill withdrawal reflex) may include short-term presynaptic or heterosynaptic facilitation of convergent pathways [50,51] or long-term cellular changes [52,53].

Although such an intrinsic-extrinsic classification of sensitization is useful, their distinction is not always clear-cut. Thus, a stimulus could sometimes induce both forms of sensitization simultaneously rather than one or the other exclusively. In the snail *Helix aspersa *tentacle withdrawal reflex, for instance, mixed intrinsic-extrinsic sensitization may be induced by a strong intrinsic stimulus when combined with inputs from the CNS [27,49].

Another anomaly to the above classification scheme is exemplified by the whole-body shortening reflex of the medicinal leech *Hirudo medicinalis *[48,54], in which a stimulus at one site of the leech body wall may sensitize the response to the same stimulus at a proximal but distinct body wall site. This phenomenon is analogous to extrinsic sensitization even though it involves only "intrinsic" stimuli and does not generalize to other loci or other sensory modalities. Similarly, in the "intrinsic sensitization" of *Aplysia *tail withdrawal reflex, activity of one tail sensory neuron during habituation training may heterosynaptically facilitate the response to a proximal but untrained tail sensory neuron [55]. Apart from the similarity of the sensitizing and test stimuli, however, such "intrinsic sensitization" is mechanistically analogous to "extrinsic sensitization" in *Aplysia *gill withdrawal reflex [51]. These seeming anomalies call for a revamping of the taxonomy for sensitization.

#### 2.2.2. Primary and secondary sensitization

The above intrinsic-extrinsic classification of sensitization is based solely on the induction process. As pointed out by Prescott [31], it is important to distinguish the *induction *and *expression *phases of sensitization. Here we propose a unified framework that rectifies the ambiguities of intrinsic and extrinsic sensitization.

**Figure 1:**
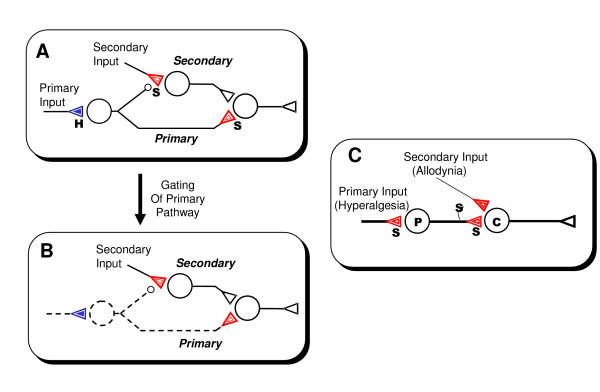
Primary and secondary sensitization and input-gating effect and their correspondence to hyperalgesia and allodynia forms of pain sensitization. **A**. Schematic illustration of habituation (H) and secondary sensitization (S) mediated by homosynaptic STD (filled inner triangle, blue) in primary pathway and heterosynaptic STP (open inner triangle, red) in secondary pathway. Open triangles denote non-adaptive excitatory synapses. Primary sensitization could occur independently of habituation through homosynaptic STP (open inner triangle, red) in primary pathway. Other primary-secondary pathway configurations of nonassociative learning are also possible [140]. **B**. Input-gating effect: upon cessation of primary input, all memory components in primary pathway are gated off abruptly and become latent. **C**. Schematic illustration of hyperalgesia and allodynia respectively as primary and secondary sensitization at peripheral (P) or central (C) sites. The pain sensation and sensitization are relieved once the stimulus ceases – a behavior that epitomizes the input-gating effect.

In keeping with the dual-process theory we refer to the stimulus that induces learning as the *primary stimulus *and the corresponding stimulus-response pathway the *primary pathway*. Further, any collateral pathway that is indirectly influenced (e.g., through heterosynaptic or presynaptic modulation) by the primary pathway is termed *secondary pathway *and the corresponding driving stimulus a *secondary stimulus*. Under this nomenclature, we define *primary and secondary sensitization *as sensitization expressed in the primary or secondary pathway, respectively, regardless of any extrinsic influences on the corresponding induction process (Fig. 1A).

This emphasis on expression instead of induction of sensitization circumvents the ambiguities in previous studies. On one hand, primary sensitization is analogous to intrinsic sensitization in that both are induced and expressed directly in the primary pathway. On the other hand primary sensitization does not exclude possible extrinsic influences as does intrinsic sensitization. Similarly, secondary sensitization is analogous to extrinsic sensitization in that both are expressed for a stimulus different than the sensitizing stimulus, but secondary sensitization is distinguished from primary sensitization by its indirect expression. Thus, secondary sensitization satisfactorily accounts for extrinsic sensitization and reconciles the seeming discrepancy with the above-mentioned anomalies [48,54,55].

#### 2.2.3. Sensitization of pain: hyperalgesia and allodynia

The above definitions of primary and secondary sensitization of sensory inputs may also shed light on the problem of peripheral or central sensitization of pain pathways following physical insults. Peripheral sensitization is mediated by noxious input-dependent release of inflammatory neuromodulators which (by activating protein kinases) increase the transduction sensitivity and excitability of the nociceptor terminal. "Classical" central sensitization is mediated by activity-dependent increases in excitability or expansion in receptive fields of nociceptive relay neurons at the superficial (lamina I) or deep (lamina V) dorsal horn of the spinal cord [56] or higher-order central sites. Inflammatory modulators increase the excitability of these relay neurons by activating protein kinases, blocking specific glycine receptor subtype [57] or upregulating specific sodium channels [58]. In recent years, other forms of central sensitization have been found which involve activity-dependent increases in synaptic efficacy of these relay neurons, with varying onset latencies and memory durations reflecting distinct transcription-dependent or -independent cellular events [59,60]. These modern forms of central sensitization have been likened to synaptic plasticity-related learning and memory [59-63]. Both peripheral sensitization and the classical or modern forms of central sensitization contribute to hyperalgesia (increased responsiveness to noxious stimuli) although their mechanisms and loci in the pain pathway may vary. In the present framework, such nonassociative and input-dependent hypersensitivity mechanisms of hyperalgesia are in perfect agreement with the notion of primary sensitization as defined in Section 2.2.2.

In contrast to hyperalgesia, which pertains to the same nociceptive input perpetuating the pain sensation, allodynia is hypersensitivity to normally innocuous inputs (such as gentle touch) secondary to a nociceptive input. A prevailing explanation of tactile allodynia is that low-threshold mechanosensitive Aβ afferents with weak synaptic connection at nociceptive relay neurons may be presynaptically or heterosynaptically sensitized by the primary nociceptive input, thus facilitating this normally silent tactile pathway (Fig. 1C) [59,64]. A similar explanation may also apply to spontaneous pain if the sensitized convergent pathway has tonic activity. Such nonassociative and activity-dependent sensitization of convergent pathway mediating allodynia lends further support for our definition of secondary sensitization as a generic mode of nonassociative learning in neural pathways (Sect. 2.2.2).

In some instances, pain sensations (especially milder types of pain) may habituate upon repeated presentation of the stimuli [65]. The pain habituation, hyperalgesia and allodynia effects of pain sensation are analogous to the habituation, primary sensitization and secondary sensitization forms of nonassociative learning.

### 2.3. Dual-process theory reconciled

#### 2.3.1. Relations to intrinsic and extrinsic sensitization

Our definitions of primary and secondary sensitization clarify the ambiguities of the dual-process theory. The original theory pertaining to an acute spinal cat preparation was predicated on a sensitization-habituation complex observed in the cat's hindlimb flexion reflex response to a repetitive electrical stimulus. The sensitization was attributed to certain interneurons presumably located in an extrinsic "state" system that was directly activated by the primary stimulus [10,11]. As such, the sensitization process on which the theory was based is neither intrinsic nor extrinsic sensitization.

By contrast, the sensitization-habituation complex of the cat hindlimb flexion reflex fits well with the notion of *primary *sensitization and habituation. Rather than mediated by an extrinsic state system as originally proposed, sensitization induced by a repetitive primary stimulus could be expressed in the primary pathway(s) as with habituation, thus evidencing primary sensitization. Indeed, as demonstrated in the frog spinal reflex, primary sensitization and habituation could occur even across the same synaptic junction [38].

Another instance of sensitization in the cat hindlimb flexion reflex was observed when a strong stimulus was delivered at skin sites near the primary stimulus that induced the dual-process response sensitization-habituation. The resulting response sensitization differed from dishabituation in that it decayed spontaneously regardless of the continuance/discontinuance of the primary stimulus [10]. However, this form of sensitization is clearly distinguishable from the first, which was elicited by the primary stimulus itself. Rather, it resembles secondary sensitization as defined in Section 2.2.2 (but with the primary and secondary pathways reversed) in that it was induced by a separate stimulus, perhaps via heterosynaptic or presynaptic facilitation. Thus, primary and secondary sensitization effectively account for all experimental data that formed the cornerstone of the dual-process theory.

Another experimental paradigm that motivated the dual-process theory was the rat acoustic startle response [10,66]. As with the cat hindlimb flexion reflex, primary and secondary sensitization are evident in this reflex in the form of complex response sensitization-habituation to a repetitive primary (auditory) stimulus and a subsequent, spontaneously-decaying sensitization triggered by a secondary (visual) stimulus. Thus, the present definitions of habituation and primary/secondary sensitization provide a unified theoretical framework that reconciles the dual-process theory and varying definitions of sensitization.

#### 2.3.2. Secondary sensitization as "generalization of sensitization"

The notion of secondary sensitization also rectifies another archaic conjecture of the dual-process theory, namely, the so-called "generalization of sensitization" where sensitization to one input may supposedly spread to other inputs [9,10]. This conjecture is in actuality an oxymoron as the generalization of intrinsic sensitization simply amounts to extrinsic sensitization, both being instances of secondary sensitization.

#### 2.3.3. Dishabituation as primary or secondary sensitization

According to the dual-process theory, the so-called "dishabituation" effect was neither a disruption of habituation nor an independent process in itself, but rather, an instance of sensitization superimposed on habituation – such that the dual process of habituation and sensitization would adequately account for all incrementing and decrementing behavioral responses. Although this view was later challenged by studies of *Aplysia *gill- and siphon-withdrawals, which revealed certain subtle differences between dishabituation and sensitization at the behavioral and cellular levels [67,68], such discrepancies were subsequently found to be attributable to an interaction between habituation and inhibition in some modulatory pathways [69,70]. Thus, dishabituation may represent a form of sensitization that is gated by habituation (see Sect. 4.3.2).

Most previous studies of dishabituation used a secondary stimulus to reverse habituation. In a recent model of tail-elicited siphon withdrawal in *Aplysia *[69,71], however, dishabituation is expressed in reflex pathways both ipsilateral or contralateral to the primary stimulus even though habituation and sensitization are expressed only in the pathway ipsilateral to the primary stimulus. This finding is consistent with the notion of primary and secondary sensitization, in that dishabituation could be expressed in both primary and secondary pathways rather than confined to the primary pathway as suggested by the dual-process theory.

Thus, the present framework brings into harmony a body of confounding observations relating to intrinsic, extrinsic and anomalous sensitization, generalization of sensitization and dishabituation, which are otherwise incongruous with the dual-process theory.

## 3. Desensitization: a novel form of nonassociative learning

The above framework of habituation and primary/secondary sensitization is complementary to a new mode of nonassociative learning called *response desensitization*. In the following, we review the experimental evidence of response desensitization and show how this novel concept may yield new insights to some sensory remapping behaviors such as phantom sensation and drug addiction.

### 3.1. Desensitization as nonassociative learning

#### 3.1.1. Desensitization as secondary habituation

**Figure 2:**
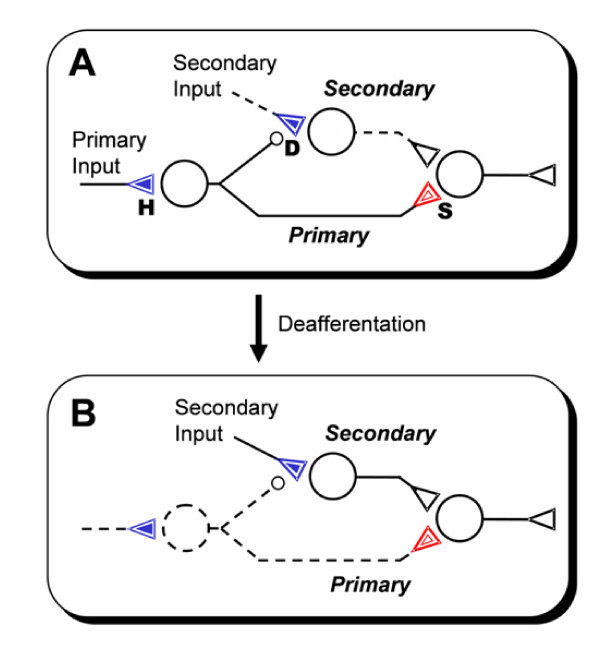
Response desensitization and phantom sensation. **A**. Schematic illustration of desensitization (D) in relation to habituation (H) and primary sensitization (S). The secondary pathway is desensitized by heterosynaptic STD (filled inner triangle, blue) secondary to the primary stimulus, leaving it dormant (broken line). **B**. Phantom sensation: following deafferentation, the primary stimulus ceases and the secondary pathway is re-sensitized, producing a phantom sensation.

A corollary to the above definitions of primary and secondary sensitization is the notion of *primary and secondary habituation*. For simplicity, we abbreviate primary and secondary habituation as *habituation *and *desensitization*, respectively (Fig. 2A).

#### 3.1.2. Desensitization of descending pathways

Instances of response desensitization have often gone unnoticed because it is easily mistaken for "habituation". An example is the crayfish tail-flip escape response to repetitive primary afferent activations, which exhibited tonic GABAergic inhibition via a descending pathway to the motor circuitry [72]. Desensitization as a new mode of nonassociative learning was first formalized in studies of the rat Hering-Breuer reflex, which evidenced a decrementing response adaptation secondary to habituation upon sustained application of a primary (vagal) stimulus and a short-term memory of the adaptation upon termination of the primary stimulus [3]. The secondary adaptation component was selectively abolished by pontine lesion or pharmacological blockade of NMDA receptor-gated channels, suggesting STD of tonic excitation (or STP of tonic inhibition) of some descending ponto-medullary pathway [73,74]. The habituation-desensitization paradigm exemplified by these animal models is in contrast to the purported habituation-sensitization dual process that has permeated previous studies of nonassociative learning.

#### 3.1.3. Desensitization as generalization/transfer of habituation

Indeed, response desensitization has long lurked under the dual-process theory as the putative "generalization of habituation" [75] or "transfer of habituation" [76,77] (see Sect. 2.1.2), sometimes also called "extrinsic habituation" [37]. Generalization of habituation *between sensory modalities *is best illustrated by the *Aplysia *siphon and gill withdrawal reflex, in which habituation training at one sensory site (gill) may transfer to an untrained site (siphon) through heterosynaptic modulation (e.g., heterosynaptic depression or inhibition [78]) via the peripheral nervous system [77] or some perceptron-like parallel processing [79]. Generalization of habituation *between sensory sites *is seen in the escape swim of the marine mollusk *Tritonia diomedea *[80] and the shortening reflex of the medicinal leech *Hirudo medicinalis *[48,54], where response habituation elicited at one body site may transfer to an untrained site.

The putative generalization/transfer of habituation is converse to the secondary sensitization in similar animal models (albeit with differing stimulus intensity or type). In particular, the heterosynaptic inhibition in *Aplysia *is mechanistically opposite to the heterosynaptic facilitation that is thought to contribute to the extrinsic sensitization of its gill- and siphon-withdrawal reflex [51]. Therefore, generalization/transfer of habituation is operationally and mechanistically analogous to secondary sensitization but with differing response polarity and activation threshold. As such, it represents a distinct form of nonassociative learning in its own right. These observations lend further support for response desensitization as a *bona fide *mode of nonassociative learning rather than an extension of habituation.

### 3.2. Desensitization and referred pain sensations

#### 3.2.1. Somatosensory remapping

The present notion of response desensitization as a new mode of nonassociative learning may shed light on the enigmatic "referred phantom sensation" (such as phantom pain) experienced by some amputees [46,81,82]. Recent findings have linked such phantom sensations to remapping at cortical [83] and thalamic or sub-thalamic levels [84] such that the deprived primary pathway is referred to a separate pathway with distinct receptive field and an expanded central representation that invades the original primary representation. A possible mechanism of such remapping is collateral sprouting (a rather slow process); another prevailing hypothesis is that such referred pathway may be preexisting but latent, and are unmasked after deafferentation [85,86] presumably by disinhibition [86-89].

However, disinhibition is a fast neurotransmission process that may take effect rapidly. Although rapid somatosensory reorganization post-deafferentation (within minutes) has been reported [90,91], amputated subjects generally do not experience phantom sensations until much later. As pointed out by Chen, Cohen and Hallet [92], the mechanisms of nervous system reorganization following injury may differ depending on the timeframe. The timeframe of phantom sensations (reportedly in hours or days) [93,94] does not appear to match those of sprouting (in weeks or months) or disinhibition (in seconds and minutes), suggesting that other mechanisms of remapping might be involved.

#### 3.2.2. Two-tier learning model of phantom sensation

As an alternative hypothesis, we suggest that phantom sensation might result from unmasking of latent somatosensory pathways through learning and memory instead of (or in addition to) disinhibition, perhaps by means of synaptic plasticity such as long-term potentiation (LTP) (which has been implicated in the reshaping of cortical motor maps [95]). In support of this hypothesis, recent evidence indicates that somatosensory reorganization associated with perceptual learning in human subjects may occur within a timeframe of hours of training and may be controlled by similar basic mechanisms that underlie NMDA receptor-dependent synaptic plasticity such as LTP [96]. Furthermore, similar molecular mechanisms that underlie NMDA receptor-dependent homosynaptic LTD in the rat visual cortex have been linked to the characteristic visual impairment resulting from hours of monocular deprivation during early postnatal life [97,98]. These recent findings point to a possible role for synaptic plasticity such as LTD/LTP in the masking/unmasking of somatosensory pathways before and after deafferentation.

In keeping with the notion of unmasking of preexisting pathways, we propose a *two-tier nonassociative learning model *of somatosensory organization, with the primary and referred sensations being mediated by a primary pathway and a latent *surrogate pathway*, respectively (Fig. 2B). The primary and surrogate (secondary) pathways are functionally equivalent to the primary and secondary pathways of nonassociative learning (Fig. 1A). In contrast to the inhibition/disinhibition hypothesis of unmasking, the present theory postulates that the surrogate pathway may be normally *desensitized *and, hence, rendered ineffective by the primary pathway. Deafferentation abolishes ("gates off", see Sect. 4.1) the primary pathway and its sensory dominance, allowing the intact surrogate pathway to strengthen over time through synaptic plasticity processes such as LTP. The resultant sensitization effect unmasks the surrogate pathway, giving a phantom sensation. In particular, if the primary pathway is part of a pain pathway then the referred phantom sensation may give rise to phantom pain if the surrogate pathway or its referred central representation in the pain pathway is hypersensitized.

The above model suggests a new perspective to the neural reorganization that reportedly underlies phantom sensations. In those patients, recordings in the thalamic region that normally respond to the missing limb revealed new receptive fields on its stump; microstimulation of this remapped thalamic region evoked phantom sensations of the missing limb, including phantom pain [84]. These findings suggest that the thalamic representation of the amputated limb was remapped to a surrogate pathway from the stump of the missing limb, presumably via learning. This two-tier learning model of somatosensory remapping based on the general theory of nonassociative learning provides a coherent explanation of referred phantom sensation and phantom pain and related experimental observations in relation to a general class of synaptic plasticity and nonassociative learning processes widely reported across animal phyla from invertebrates to humans.

#### 3.2.3. Capsaicin sensitization and "desensitization"

The response desensitization as defined above is distinct from the desensitization of nociception associated with certain irritants such as capsaicin, the pungent chemical in red chili pepper. In human subjects, the burning/pricking sensation elicited by oral capsaicin typically intensifies with its repeated applications at an ISI of ~1 min, a hyperalgesic effect that is akin to response sensitization. However, following a hiatus of several minutes reapplication of capsaicin elicits a much weaker sensation. This latent refractory process has been called "desensitization" by some authors [47,99] in analogy to desensitization of nociceptive vanilloid receptors, which are generally thought to mediate the pungency of capsaicin [100].

The waxing and waning of the pungency following repeated capsaicin application is reminiscent of the sensitization-habituation dual process of nonassociative learning [10,37]. As such, the refractory response to capsaicin following sensitization training should be a classic case of habituation in the primary nociceptive pathway instead of "desensitization". The successive increase and decrease of capsaicin pungency on varying timescales indicates that the habituation component develops more slowly but lasts longer than sensitization. If so, a weaker sensitization effect should unmask the progressive development of habituation during behavioral training. Indeed, repetitive oral application of other irritants such as nicotine, menthol, zingerone or mustard oil elicits sensations that decline successively across trials [101-104], evidencing response habituation with weak or no sensitization.

It has been suggested that capsaicin sensitization may be mediated by an increase in excitability of peripheral nociceptors or central relay neurons, or spatial recruitment of vanilloid receptors in nociceptor endings [99]. These hypothesized cellular mechanisms are consistent with primary sensitization (as defined in Sect. 2.2.2). On the other hand, capsaicin sensitization has been shown to promote hypersensitivity to and aftersensations of other pain stimuli applied to the affected site [105]. This secondary hyperalgesic effect is indicative of secondary sensitization or central sensitization involving secondary nociceptive or allodynic pathways, perhaps via wide dynamic range neurons in spinal dorsal horn [56].

### 3.3. Desensitization and drug addiction

It is well-known that repeated drug administrations may result in drug tolerance and/or sensitization [106], which are attributable to the dual process of response habituation and sensitization [107-109]. The notion of response desensitization presently proposed adds a new dimension to the understanding of the behavioral mechanisms of drug tolerance and, indeed, of drug craving and addiction itself.

#### 3.3.1. Sensitization models of craving

Craving plays an important role in the pathogenesis of many addictive disorders (such as alcohol, nicotine, narcotic or other psychostimulant drug dependencies) but its mechanism has remained unclear [110,111]. Current models of craving (for overviews, see [112,113]) ascribe this psychophysical drive to certain cognitive or neuroadaptive processes such as behavioral sensitization – a phenomenon characterized by enhanced psychomotor and motivational effects of an addictive drug along with increased midbrain dopamine neurons reactivity upon repeated drug applications [114,115]. Recent evidence suggests a possible link between behavioral sensitization and LTD of AMPA receptor-mediated synaptic transmission in the nucleus accumbens [116]. Although behavioral sensitization is not tantamount to craving, it is often thought to induce compensatory homeostatic or incentive-motivational adaptations which, in turn, could incubate craving or "pathological wanting" that may be rekindled by stress or drug cues during prolonged abstinence [117-121].

#### 3.3.2. Desensitization model of craving

**Figure 3:**
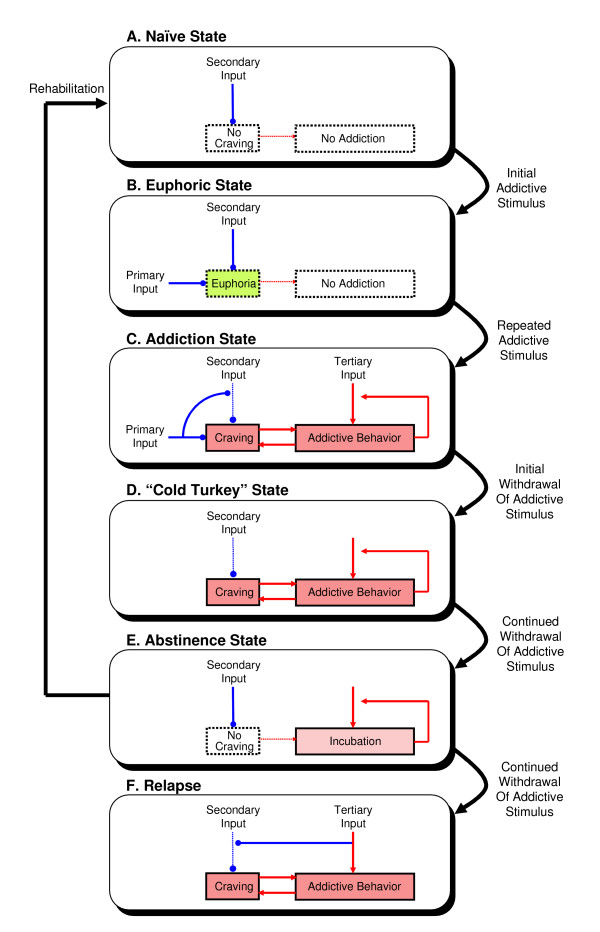
Graphical depiction of a theoretical model of addiction and recovery. (A) In the naïve state, an intrinsic secondary input suppresses craving via an inhibitory pathway. (B) Upon initial exposure to an addictive stimulus, the craving center is further inhibited by the primary pathway resulting in a feeling of euphoria. (C) With continued exposure to the addictive stimulus, the secondary pathway is desensitized by the primary pathway (with possible habituation in the primary pathway not shown), resulting in craving and the onset of addictive behavior. The latter can lead to more craving via a positive feedback vicious cycle as well as mobilization of a tertiary process that can independently perpetuate the addictive behavior. (D) Sudden withdrawal of the addictive stimulus precipitates a state of "cold turkey" characterized by enhanced craving due to the loss of the primary input, continued desensitization of the secondary pathway and continued positive feedback from the addictive behavior. (E) Sustained abstinence will allow resensitization of the secondary pathway and temporary relief of craving. The addictive behavior subsides, but the tertiary process is still lurking and intensifying. Complete rehabilitation to the naïve state (A) calls for extirpation of the tertiary process. (F) Otherwise, reactivation of the tertiary pathway by contextual cues, memory or stress could once again desensitize the secondary pathway, triggering a relapse.

In contrast to previous sensitization models of craving during relapse, we propose a desensitization model of craving during the onset of addiction, as follows (Fig. 3). Central to our model is the notion that craving of any kind may represent an innate (rather than acquired) instinct, not fundamentally different than basic instincts such as thirst, hunger, sex, and yearning for love or happiness, etc. However, unlike ordinary psychophysical drives that are critical for animal survival or procreation and are expressed at birth or during puberty, craving for substance of abuse is functionally deleterious (hence "pathological") and hence its expression is likely to be repressed through evolution. In a naïve (or "innocent") state, pathological craving may be inhibited intrinsically by certain tonic central inputs that promote self-restraint (or self-reward) – presumably via some midbrain dopaminergic or glutamatergic pathways – hence keeping craving and addiction in check. Exposure to an addictive drug may disrupt this equilibrium state and arouse addiction in multiple possible ways. Firstly, it activates (i.e., "gates on", see Sect. 4.1) a normally-latent primary sensory pathway that serves to relieve craving and evoke gratification in addition to the central (secondary) craving-inhibiting pathways, thus producing an immediate euphoric effect. Secondly, activity in the primary pathway may in turn desensitize the secondary craving-inhibiting pathways, thus debilitating the brain's natural defensive against addiction. This critical step may correspond to the "loss of inhibitory control in decision making" against addiction suggested by some investigators [118]. Thirdly, desensitization in the secondary pathways (together with possible habituation in the primary pathway) may cause increasing tolerance to the drug, hence drawing higher-and-higher dosages in order to relieve craving or regain euphoria, further deepening the addiction. Finally, abrupt abstinence in a desensitized state may unleash the craving and precipitate any accompanying withdrawal symptoms, which may subside over time as the reward system gradually re-sensitizes.

The above model predictions depict the early drug-induced degeneration of the brain's reward system from a naïve state to a desensitized state and the subsequent recovery during abstinence. However, once exposed to an addictive stimulus the brain may not be totally "innocent" anymore in that the reward system may begin to give way to other, non-reward related hysteretic mechanisms which may ensue even after recovery. For example, repeated drug exposures may mobilize other neuroadaptive processes, such as behavioral sensitization, which promote relapses (see above). Also, a craving-driven addiction could later turn into a completely craving-free habit or even compulsion [122-124], perhaps via the dynamic modulation of some cortical-basal ganglia circuits [125]. These tertiary hysteretic processes could perpetuate the drug-seeking behavior independent of dopamine-mediated reward [126,127], with or without provoking craving [128,129]. On the basis of these observations, we suggest that desensitization of secondary craving-inhibiting pathways and sensitization of tertiary hysteretic pathways may underlie the acquisition and maintenance of addiction behavior, respectively, such that their sequential inductions upon the first encounter with an addictive drug create a watershed effect that irreversibly usurps the brain's built-in self-restraint mechanism to stave off addiction (Fig. 3).

#### 3.3.3. Context-dependent habituation and desensitization

In contrast to habituation and primary sensitization (the quintessential dual process of nonassociative learning), desensitization and secondary sensitization could also involve associative training by primary and secondary stimuli. Some classic models of nonassociative learning such as *Aplysia *siphon and gill-withdrawal reflex are known to demonstrate classical conditioning, which shares similar cellular and molecular mechanisms with nonassociative learning [130-132]. A variant of conventional habituation called context-dependent habituation has been shown in some animal models such as the nematode *C. elegans *[133] and the crab *Chasmagnathus *[134], where the retention of habituation after training is also influenced by certain environmental cues. The dependence on environmental cues indicates that the primary stimulus-response pathway is not merely habituated by the primary input but likely also desensitized by certain context-dependent sensory inputs in an associative manner.

Such context-dependent learning effect has important implications in certain addictive disorders, where environmental cues are known to promote drug-dependent behavioral sensitization and relapses [121,135]. Hence, environmental cues may serve as a conditioned stimulus to certain tertiary pathways which, when activated, may independently desensitize the secondary craving-inhibiting pathways during abstinence (Fig. 3) in a manner analogous to the conditioned drug response in associative learning [136,137]. Further studies are needed to elucidate the possible role of associative learning in the induction of secondary sensitization and desensitization in these experimental models.

## 4. Temporal filtering by nonassociative gating

The notion of primary and secondary sensitization introduced in Section 2 underscores a novel behavioral paradigm we call *nonassociative gating*. Several forms of nonassociative gating have been identified that provide computational capabilities complementary to nonassociative learning.

### 4.1. Input gating

#### 4.1.1. Input gating and memory recall

An important property of neurotransmission memory is that it is discernible only during recall, i.e., when the pathway is activated by a stimulus eliciting combined reflex and memory responses. Once the activation ceases, the corresponding memory becomes latent and unobservable. We call this an *input gating *effect, namely an on-off switching of neurotransmission memory by the stimulus itself (Fig. 1B). Thus, a primary stimulus that induces learning and memory in the primary pathway may simultaneously recall the memory by "gating" it on. Conversely, memory in the primary pathway is automatically gated off once the primary stimulus disappears and thus any residual response must reflect persisting activity in the secondary pathway [2,138].

For instance, deafferentation effectively gates off the primary pathway (Sect. 3.2.2). Another example is the phenomenon of pain sensitization (Fig. 1C). In hyperalgesia, increased pain sensation due to peripheral or central sensitization is elicited when a noxious stimulus is applied but these effects are promptly relieved (gated off) once the stimulus is removed. In the case of addiction, initial exposure to an addictive drug gates on a normally latent craving-suppressing primary pathway thereby setting off the addiction vicious cycle (Fig. 3).

#### 4.1.2. Maximum interstimulus interval

The notion of input gating has important implications in determining the maximum ISI for nonassociative learning experiments. With a repetitive stimulus the primary memory is simultaneously induced and recalled at successive stimulus episodes but is gated off in between. Therefore, for optimal memory recall an ISI should be no longer than the decay time of the primary memory. This maximum ISI condition for training is tacit in studies of nonassociative learning reported in the literature.

#### 4.1.3. Primary and secondary memory

The activity-dependent and pathway-specific properties of input gating make it a useful behavioral marker for memory in the primary pathway vs. those via the secondary pathway, hereinafter referred to as *primary and secondary memory*, respectively. This marker readily distinguishes nonassociative learning modes mediated by the primary and secondary pathways. Thus, habituation and desensitization are readily distinguished by the absence/presence of a STD memory trace in the resultant behavioral response, whereas primary and secondary sensitization are distinguished by corresponding absence or presence of a STP memory trace. These criteria have been successfully applied to the experimental classifications of primary and secondary memory in the rat respiratory chemoreflex and mechanoreflex [2,3,74,138-140].

### 4.2. Output gating

#### 4.2.1. Output gating and refractory period

Memory recall requires not only an enabling input but, also, an observable output. In some sensory modalities such as olfaction and vision, the output of nonassociative learning is registered continuously as a sensory percept in the brain without fail and thus the memory trace is gated only by the input. In other sensory modalities, however, the behavioral output may be registered as discrete motor response (or other effector response) with a definite refractory period. If so, the memory trace may be gated off during the refractory of the output as well. We call this an *output gating *effect in contradistinction to input gating.

#### 4.2.2. Minimum interstimulus interval

The notion of output gating has important implications in determining the minimum ISI for nonassociative learning experiments. For example, in the classic *Aplysia *gill-withdrawal reflex a strong tactile stimulus to the siphon may produce a strong and long-lasting gill response which, if unabated, may mask the responses to subsequent stimuli [141]. In this case, the minimum ISI for producing a demonstrable habituation effect is limited by the refractory period of the gill response. Conversely, a behavioral system with negligible refractory in the effector would require little or no ISI. This minimum ISI condition is tacit in studies of nonassociative learning reported in the literature.

### 4.3. Extrinsic gating

#### 4.3.1. Phase-dependent gating

**Figure 4:**
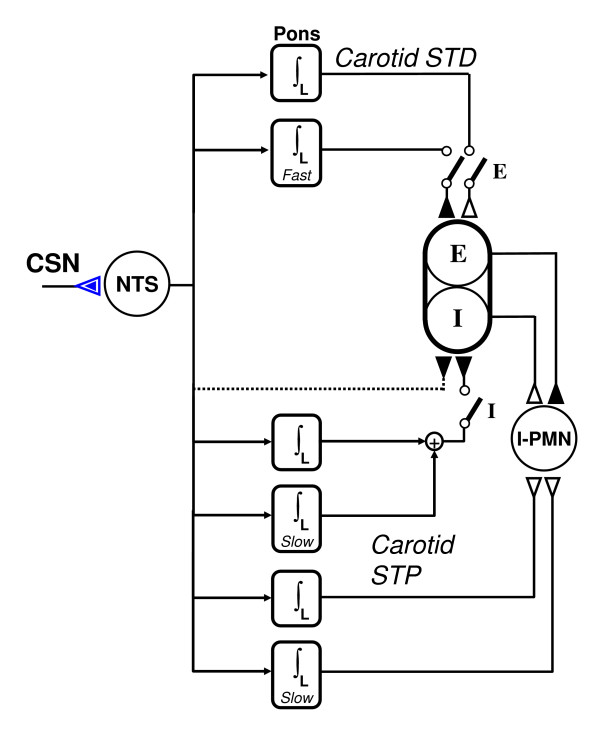
Phase-dependent gating and primary/secondary (monophasic/biphasic) integration and differentiation mechanisms as exemplified by the carotid chemoreflex modulation of respiratory rhythm in rats. The chemoafferent input from the carotid sinus nerve (CSN) is habituated by a monophasic (input gated) differentiator in the nucleus tractus solitarius (NTS). The output from NTS is relayed by parallel pathways to I (inspiratory) or E (expiratory) neurons of the respiratory pattern generator or to I-PMN (inspiratory premotor neuron). Switches denote gating to either I or E phase. Each pathway is modulated by two biphasic integrators (∫_L _with fast or slow time constant) which either add or subtract to produce net short-term potentiation (STP) or depression (STD) effects. Adapted from [138].

In contrast to input and output gating, which are intrinsic to any stimulus-response pathway, neurotransmission gating may also arise from extrinsic factors. In particular, we define *phase-dependent gating *as the on-off switching of a stimulus-response pathway by a phasic command signal independent of the pathway's input and output. This type of extrinsic gating is exemplified by the mammalian carotid chemoreflex modulation of the respiratory rhythm in which separate STP and STD chemoreflex afferent pathways are temporally gated to either the inspiratory or expiratory phase of the respiratory pattern generator (Fig. 4). Such phase-dependent gating allows the chemoreceptor input to selectively modulate each respiratory phase in an orderly manner via separate STP or STD pathways, much like the on-off switching of two-way traffic lights at an intersection [74,138].

#### 4.3.2. Learning-dependent gating

Gating may also be triggered by activity-dependent plasticity rather than a phasic command. This type of extrinsic gating is exemplified by *Aplysia *tail-elicited siphon withdrawal reflex where a modulatory network that normally inhibits the sensitization of contralateral siphon response is relieved after habituation (see Sect. 2.3.3). Such *learning-dependent gating *has been suggested to account for the bilateral expression of dishabituation vis-à-vis ipsilateral expression of sensitization in this experimental preparation [69,71].

### 4.4. Nonassociative gating as Boolean 'toggle switch'

#### 4.4.1. Nonassociative gating: a new behavioral paradigm

Input/output gating and extrinsic gating are instances of *nonassociative gating*. As with nonassociative learning, the induction of such gating effects is activity-dependent and nonassociative, and their expressions may be intrinsic or extrinsic to the neurotransmission pathway. Furthermore, nonassociative gating displays certain computational characteristics that are complementary to nonassociative learning.

#### 4.4.2. Boolean on-off switching and temporal filtering

In input gating, the stimulus itself provides a logic 'on' signal that enables memory recall whereas in output gating, the effector response serves as a logic 'off' signal that momentarily disables or attenuates memory recall. Similarly, in phase-dependent or learning-dependent gating an extrinsic signal independent of the current input or output provides the on-off command for the memory trace. Thus, nonassociative gating operates like a Boolean toggle switch that may turn the memory trace on or off depending on the logic value of the command signal.

Alternatively, nonassociative gating may be viewed as a *temporal filter *that selectively passes or stops memory recall within specific time windows during nonassociative learning. Such signal filtering in the time domain contrasts with the signal filtering in the frequency domain by nonassociative learning.

## 5. Frequency filtering by nonassociative learning

Based on the above, we propose a theory of gated integral-differential neural computation (or low-pass and high-pass frequency filtering) by nonassociative learning. Prescott [31] has proposed a mathematical model that mimics the kinetics of habituation and intrinsic sensitization development and their interaction using linear first-order differential equations. Dragoi [142] has proposed a similar model of suppressive and facilitatory interactions during nonassociative learning but with nonlinear first-order differential equations in order to simulate the rate sensitivity property of habituation. Staddon and Higa [143] have proposed a feedback/feedforward integrator model of habituation. Shen [144] has proposed a STP model of neural integrator. The present theory differs from the previous models in that it is structurally-based and includes all four modes of nonassociative learning (Sect. 2.4.1) as well as nonassociative gating (Sect. 3.1.3), which provide a complete mathematical basis for gated integral-differential computation.

### 5.1. Definitions of neural integrator and differentiator

#### 5.1.1. Leaky integrator and differentiator

Numeric integration and differentiation are elemental calculus operations. They also underlie all temporal dynamics and kinematics phenomena in Nature. An analog integrator/differentiator is a physical process that demonstrates integral/differential input-output transformation in real time. Analog integrator and differentiator are subject to response-limiting *leakages*. Leaky integrators are commonly used in electrophysiology experiments to obtain a moving-average estimate of neuronal firing frequency called 'neurogram.'

#### 5.1.2. Integrator and differentiator response characteristics

**Figure 5:**
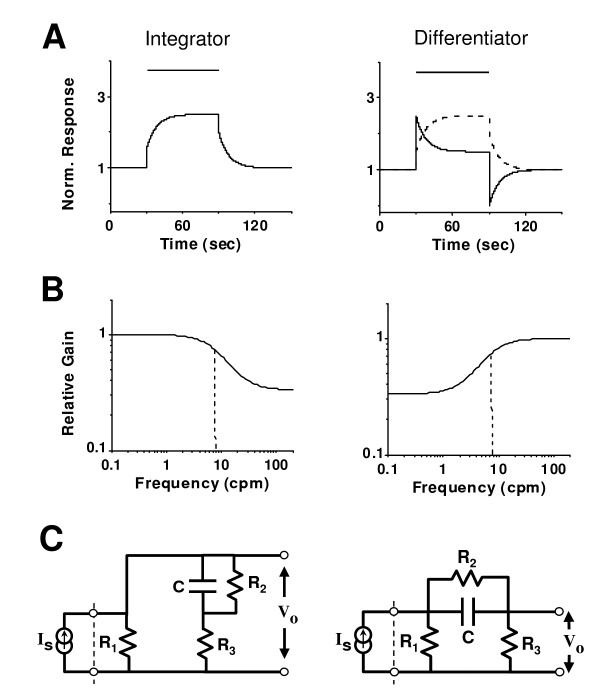
Time and frequency response characteristics of integrator (*left *panels) and differentiator (*right *panels). **A**. The temporal response of a leaky integrator to a constant-step stimulus (horizontal bar) consists of abrupt reflex increase/decrease of the response at stimulus onset/cessation followed by exponentially increasing/decaying (potentiation/afterdischarge) on/off transients. A leaky differentiator has similar reflex components but with exponentially decaying (accommodation) on-transients and rebound off-transients, which are opposite to those of an integrator (overlaying dotted lines). In both cases the off-transients may be rectified, with the response becoming monophasic (not shown) instead of biphasic. The time scales chosen are typical of oculomotor integrator and respiratory integrator. **B**. In the frequency domain, an integrator/differentiator behaves like a low-pass/high-pass filter. The pass-band in both cases is the frequency range where the neurotransmission gain (normalized to unity) is highest and relatively constant. The high and low cut-off frequencies (vertical dotted lines) of these filters are inversely proportional to the time constants of the corresponding integrator and differentiator shown in A. **C**. Examples of RC integrator (C = 3.77, R_1 _= 1.60, R_2 _= 24.0, R_3 _= 0.73) and differentiator (C = 1.0, R_1 _= 1.75, R_2 _= 24.0, R_3 _= 10.2) circuits with a current source (I_S_) input and voltage (V_0_) output. Units are arbitrary and RC values correspond to parameter values as defined in Eq. 1 (Appendix I) for integrator (*a *= 0.125; *b *= 0.125; *c *= 1; *d *= 0.5) and differentiator (*a *= 0.125; *b *= 0.125; *c *= -1; *d *= 1.5).

In the time domain, a leaky integrator's response to a constant-step input exhibits exponential saturation during on-transient and exponential decay during off-transient, whereas the corresponding response of a leaky differentiator demonstrates exponential decay from an initial overshoot and exponential recovery from rebound undershoot (Fig. 5A; Eq. 2 in Appendix I). An integrator or differentiator that sustains an off-transient response is said to be *biphasic *(or else, *monophasic*); it is said to be *inverted *if the gain is negative (Eq. 3 in Appendix I). The *dynamical order *of a compound integrator/differentiator is the number of integrators/differentiators it is composed of, and its *memory order *is the number of component integrators/differentiators that are biphasic. Under these broad definitions, a neural system that displays such integral/differential neurotransmission characteristics is called a *neural integrator/differentiator*.

#### 5.1.3. Low-pass and high-pass frequency filter characteristics

From linear systems theory [145], integrator/differentiator response characteristics in the time domain correspond to low-pass/high-pass filter characteristics in the frequency domain (Eq. 4 in Appendix I). The time constant of a leaky integrator or differentiator is inversely proportional to the cut-off frequency of the equivalent low-pass or high-pass filter, respectively (Fig. 5B). In addition to frequency filtering, a leaky integrator/differentiator also introduces phase shift (phase-lag/phase-lead) in the input-output relationship.

#### 5.1.4. Complementarities of neural integrator and differentiator

Mathematically, a neural differentiator is an *additive complement *of an integrator, i.e., the response of a differentiator is complementary to that of an integrator with similar time constants (Fig. 5A). The frequency characteristics of a low-pass and high-pass filter with matched cut-off frequencies are also complementary to one another (Fig. 5B). Indeed, combination of such complementary filters in parallel approximates an all-pass filter with constant throughput gain at all frequencies.

### 5.2. Reverberation models of neural integrator

#### 5.2.1. Reverberating neural network hypothesis

A widespread hypothesis of neural integrator is that of reverberation in a recurrent neural network. This hypothesis has been studied most extensively in two experimental models: the "afterdischarge" phenomenon in the chemoreflex control of breathing [146] and the oculomotor integrator [147-149]. The proposed mechanism involves two steps: intrinsic membrane properties of a neuron provide a trace capacitance that acts as a seed for the integrator, and positive feedbacks via a recurrent network allow continual refreshment of the seed. The network reverberation hypothesis is bolstered by the finding that the goldfish oculomotor integrator during normal saccadic movements could not be reproduced by saccade-like changes in neuronal firing induced by intracellular current injection, suggesting that the integrator effect is dependent on persistent changes in synaptic inputs [150]. It has been proposed that such a recurrent network can be made robust by certain bistable neuronal processes [151,152] or recurrent synaptic excitation with asynchronous transmitter release [153], or by external sensory error feedback [154].

#### 5.2.2. Reverberating neuronal ion-channels hypothesis

Alternatively, reverberation of excitatory activity could also occur at the single-neuron level via a cascade of membrane ion channels [155,156]. The persistent activity can be elicited bi-directionally by excitatory and inhibitory inputs in a graded fashion, similar to a biphasic integrator.

#### 5.2.3. Dendritic calcium self-amplification hypothesis

A recent mathematical model [157] posits that temporal integration in a single neuron may result from self-amplifying calcium dynamics through a cellular process called "calcium induced calcium release." According to this model, synaptic inputs modulate the regenerative propagation of calcium waves along dendritic processes, resulting in calcium-dependent currents that vary directly with the temporal sum of prior synaptic inputs. This mechanism of neural integrator is thought to be robust by virtue of the intrinsic nonlinear spatiotemporal summation of the calcium waves.

### 5.3. Nonassociative learning models of neural integrator and differentiator

#### 5.3.1. Nonassociative learning hypothesis of neural integrator and differentiator

Although the above models of neural integrator are all plausible, none of them can explain neural differentiator. In contrast, nonassociative learning is based on activity-dependent changes in synaptic efficacy [51,139,140,158] or neuronal excitability [10,159] that are well documented in single neurons. These neural mechanisms are highly stable and robust with short- or long-term memories ranging from seconds to days or months, and provide a plausible explanation of both neural integrator [2,139,144] and differentiator [2,140]. For example, synaptic STD or LTD in brainstem NTS [160-162] may provide the habituation or monophasic differentiator effects in various cardiorespiratory reflexes [3,138] (see Fig. 4).

#### 5.3.2. Primary and secondary integrator/differentiator

To a first approximation, the integrator and differentiator characteristics defined in Figure 5 are mimicked by the augmenting characteristics of primary and secondary sensitization and decrementing characteristics of habituation and desensitization, respectively. In particular, primary sensitization/habituation represents a *primary integrator*/*differentiator *with monophasic characteristics whereas secondary sensitization/desensitization represents a *secondary integrator*/*differentiator *with biphasic characteristics (see Appendix II for mathematical details).

The monophasic characteristics of primary sensitization and habituation are due to input gating (Sect. 3.1). The biphasic characteristic of desensitization [3,72] may be ascribed to tonic activity in the secondary pathway, which provides a continual recall of the secondary memory (Sect. 2.5). In contrast, in secondary sensitization the roles of the primary and secondary pathways are often reversed (sensitization in the primary pathway induced by a strong secondary stimulus), and thus a biphasic integrator response would be sustained by continued repetitive application of the primary stimulus itself. Figure 4 shows examples of primary and secondary integrator and differentiator demonstrated in the mammalian carotid chemoreflex pathways [138].

Although monophasic integrator and differentiator are driven directly by the primary input, biphasic integrator and differentiator require a secondary input with persistent activity. The latter could come from reverberations at the neuronal or neural network levels in central neurons [163]. Alternatively, it may come from tonic central or peripheral inputs. For example, tonic secondary inputs to the biphasic integrators in the carotid chemoreflex pathways (Fig. 4) or vagal Hering-Breuer reflex may derive from central chemoreceptors, which provide persistent activation of the respiratory pattern generator and its afferent pathways [74].

**Figure 6:**
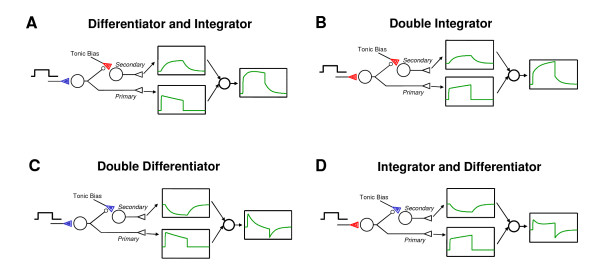
Compound neural integrator and differentiator models. Boxes show model simulations in arbitrary units and parameter values. Conventions are same as Fig. 1. **A**. Primary differentiator-secondary integrator is realized by activity-dependent habituation-sensitization in corresponding primary-secondary pathways. Step application of primary input at a constant firing rate (inset) induces synaptic STD and STP in primary and secondary pathway, respectively, resulting in temporal differentiation and integration of the transmitted signals (lower and upper boxes). Resultant response (last box) at output neuron shows a compound differentiator-integrator characteristic. **B**. A double integrator may arise from STP in both primary and secondary pathways. **C**. A double differentiator is similar to a double integrator but with STD instead of STP in both pathways. **D**. A primary integrator-secondary differentiator can be realized in a similar fashion with a STP-STD combination. Structurally, primary differentiator (**A**, **C**) and secondary differentiator (**B**, **D**) are comprised of the primary reflex in conjunction with an inverted primary and secondary integrator, respectively, demonstrating the complementarities of integrator and differentiator. See text and Appendix.

According to the complementary relationships of neural integrator and differentiator (Sect. 5.1.4), a secondary differentiator is the combination of two separate processes: a primary reflex and an inverted secondary integrator (Fig. 6C, 6D). Similarly, a primary differentiator is comprised of a primary reflex and an inverted primary integrator (Figs. 6A, 6C). Thus, a primary or secondary neural differentiator is realized by nonassociative learning as the *difference *(antagonistic excitation-inhibition combination) between a primary reflex and a primary or secondary neural integrator.

#### 5.3.3. Second-order integrators/differentiators

The four basic modes of nonassociative learning – habituation, desensitization, primary sensitization, secondary sensitization – constitute a complete orthogonal (non-redundant) mathematical basis that empowers the brain to perform basic integral-differential calculus of any dynamical and memory orders. In particular, an integrator and differentiator may combine to form an integrator-differentiator pair with second-order dynamics and frequency characteristics. The simplest example is a primary integrator-differentiator pair in the form of a sensitization-habituation complex produced by repetitive application of a primary stimulus, as demonstrated in the classic hindlimb flexion reflex of the spinal cat or the rat acoustic startle reflex shown in the dual-process theory [9-11]. The combined primary integrator-differentiator pair acts like a band-pass or band-stop filter, which selectively admits or rejects afferent inputs that are fluctuating around certain mid-frequencies.

Nonassociative learning in the primary and secondary pathways may also work in tandem to form second-order integrator-differentiator pairs. Four different combinations are possible. Habituation in conjunction with secondary sensitization gives a primary differentiator – secondary integrator pair (Fig. 6A). This is similar to the primary integrator-differentiator pair in the acoustic startle reflex but with a secondary memory. Similarly, concurrent primary and secondary sensitization results in a second-order low-pass filter in the form of a primary integrator and secondary integrator (Fig. 6B). A second-order differentiator/high-pass filter is formed by habituation in the primary pathway and desensitization in the secondary pathway (Fig. 6C), as demonstrated in the Hering-Breuer reflex or carotid chemoreflex modulation of expiratory duration in the rat [2,3,74,140]. Finally, it is conceivable that sensitization-desensitization in the primary-secondary pathway may give rise to a second-order integrator-differentiator pair (Fig. 6D) with band-pass or band-stop filter characteristics similar to those in Figure 6A. Examples of second-order integrator and differentiator are shown in Figure 4 for the mammalian carotid chemoreflex pathways [138].

## 6. Nonassociative learning and brain intelligence

In addition to performing kinematic transformations, nonassociative learning may also contribute to the integral-differential calculus and Boolean logic computations that are basic to brain decision processes. These neural integrators, differentiators and logic operators provide some of the basic building blocks of brain intelligence.

### 6. 1. Intelligent roles of neural integrator and differentiator

#### 6.1.1. Neural integrator: possible roles in sensory defensiveness, alarm reaction and sensorimotor instability

Behaviorally, a neural integrator can boost an animal's responsiveness to a recurrent noxious stimulus and (by cross-modal transfer) to other inputs even after the primary stimulus has ceased. The resultant heightening and widening of vigilance put the animal on the alert once this self-defense mechanism is triggered. This 'alarm reaction' instinct sets one free to economize and relax (by staying idle and calm) most of the time until fear-arousing episodes (e.g., terrorist attacks) set in. On the other hand, inordinate sensitization of the primary or secondary pathways could result in hypersensitivity to innocuous sensory stimuli. This mechanism is compatible with certain forms of sensory integration dysfunction such as sensory defensiveness [164] or nonassociative fear or anxiety toward impending adverse stimuli [165].

In sensorimotor control, temporal integration of error feedback may help to minimize the resultant steady-state error. On the other hand, low-pass filtering of the feedback signal may introduce excessive phase lags (phase delays) that tend to destabilize closed-loop control [145]. It has been suggested that spontaneous oscillations of sensorimotor regulation may develop with increased delays in sensory feedback [166-168].

#### 6.1.2. Neural differentiator: possible roles in selective attention, central resetting, sensory self-organization and fail-safe compensation

Functionally, a neural differentiator is a high-pass filter that preferentially admits time-varying signals, rejecting any DC biases that tend to saturate or suppress neurotransmission. This high-pass filtering effect allows the animal to automatically recalibrate the sensitivities of the primary and secondary pathways against varying background activities thereby extending the dynamic range of the stimulus-response relationship. Thus, a sustained primary input (e.g., hypertension or bronchopulmonary afferent hyperactivity) may induce compensatory habituation-desensitization or "central resetting" of primary and secondary pathways [160,169,170], whereas abolition of the primary input (e.g., due to impairment of sensory receptors or afferent pathways) may elicit compensatory dishabituation of the primary pathway and re-sensitization of the secondary pathway. As such, the secondary pathway provides a reserve surrogate or backup for the primary pathway should it ever fail. Such sensory self-organization provides a fail-safe mechanism for optimal compensation against hyper- or hypo-activity of afferent feedback in sensorimotor systems [2,171,172].

Another useful function of habituation and desensitization is to tune out repetitive inputs that prove to be innocuous, thus allowing selective attention to potentially important inputs [173-175]. Failure to do so may lead to sensory defensiveness in some individuals [164,176] and in patients with autism [177], as well as nonassociative fear and anxiety [165] or other forms of hyper-reactivity. On the other hand, because habituation and desensitization tend to suppress persistent afferent inputs, their overexpression may have deleterious effects in certain sensorimotor reflexes. For example, abnormal expression of LTD in the NTS of newborn mice devoid of functional NMDA receptors is implicated in the progressive respiratory failure and early death in these mutant animals [162].

#### 6.1.3. Compound neural integrator-differentiator: possible roles in novelty detection and selective attention

A compound integrator-differentiator/differentiator-integrator is functionally equivalent to a band-pass/band-stop filter that preferentially admits/rejects inputs whose temporal variability falls within some intermediate frequency band or time scale. For example, the acoustic startle reflex is sensitized by continuous background noise or novel inputs but may habituate on discrete repetitive tones [66]. Such a combined sensitization-habituation or sensitization-desensitization response pattern allows maximal vigilance to unexpected (and potentially alarming) inputs over mundane and insipid ones, thus sharpening novelty detection and selective attention [178]. On the other hand, excessive band-selective filtering may lead to paranoia; indeed, increased sensitization and reduced habituation are trait markers of patients with schizophrenia [179]. The possible role of this band-selective filtering mechanism in other sensory novelty-detection tasks such as dynamic predictive coding of unexpected visual information by the retina [180], or more complex selective attention tasks such as selective visual attention [181][182], deserves further study.

### 6.2 A sensory firewall for Cartesian mind-body internal model adaptation

**Figure 7:**
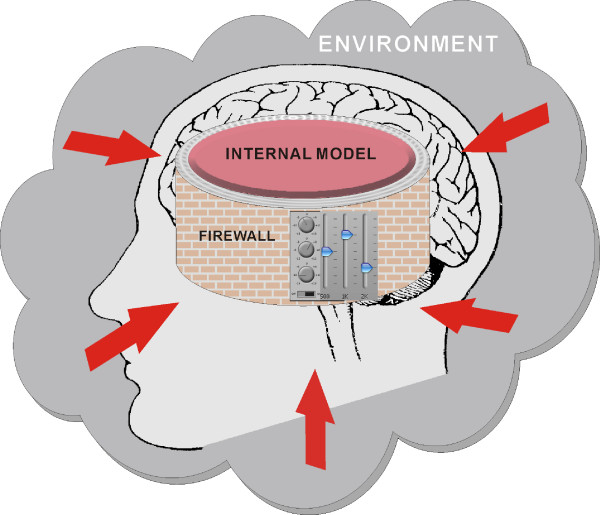
Nonassociative learning as a sensory firewall that constantly screens all environmental inputs and decides their saliency and priority for internal model adaptation in mind-body interaction. This high-pass, low-pass or band-pass/band-stop signal filtering action is analogous to tone control in an audio system.

The array of low-pass, high-pass and band-pass/band-stop frequency filtering effects of nonassociative learning, together with the associated Boolean logic temporal filtering effects of nonassociative gating, provide a finely-tuned intelligent "firewall" that continuously screens all incoming signals into actionable and non-actionable categories in order to prioritize (Fig. 7). This firewall mechanism shields the mind from the vast amounts of inundating sensory information that constantly compete with one another for attention, and spares it the trouble of having to respond to every tingling except the most salient ones. The triage process not only helps to preserve mental sanity but also conserve physical energy, both of which are important for survival. On the other hand, breakdown of the nonassociative learning processes may result in mistuning of the firewall and hence, distortions in the sensory percept (much like the frequency distortions heard in an audio system with unequal tone control). Nonassociative learning therefore plays an important role in balancing the sensory inputs. This could be the first step in the brain's putative ability of creating explicit internal models of the environment [183][184], as implicit in René Descartes' mind-body interactionism [185].

## 7. Conclusion

A general theory of nonassociative learning comprised of habituation, sensitization and desensitization in primary or secondary pathways has been presented. The defining phenotypes of these varying modes of nonassociative learning are their distinct integral-differential computation capabilities, which are shown to correlate with many intelligent or maladaptive brain behaviors. In addition, the notion of nonassociative gating with intrinsic Boolean logic computation capability has been introduced as a basic behavioral paradigm that may act independently or in tandem with nonassociative learning. Together, nonassociative learning and nonassociative gating constitute an intelligent firewall that constantly triages vast amounts of sensory information into actionable and non-actionable categories in order to prioritize. This unified framework of nonassociative learning and nonassociative gating sheds new lights on the ultra secrets of brain intelligence and brain disorders. The underlying functional and structural organization principles [186] are shown to be generally applicable to a wide variety of brain systems across animal phyla and sensory modalities in health and in disease states. These system-level principles are fundamental to a systems medicine approach [187][188] to the management of human health and disease at the organ, organism and community level.

## APPENDIX I. Integrator and differentiator equations

A first-order leaky integrator or differentiator (Fig. 5) is described by the following equations:

x˙
 MathType@MTEF@5@5@+=feaafiart1ev1aaatCvAUfKttLearuWrP9MDH5MBPbIqV92AaeXatLxBI9gBaebbnrfifHhDYfgasaacH8akY=wiFfYdH8Gipec8Eeeu0xXdbba9frFj0=OqFfea0dXdd9vqai=hGuQ8kuc9pgc9s8qqaq=dirpe0xb9q8qiLsFr0=vr0=vr0dc8meaabaqaciaacaGaaeqabaqabeGadaaakeaacuWG4baEgaGaaaaa@2E2E@ = -*ax *+ *bu* (1a) 

*y *= *cx *+ *du *    (1b)

where *y, u *are the output and input of the integrator or differentiator, respectively; *x *and x˙
 MathType@MTEF@5@5@+=feaafiart1ev1aaatCvAUfKttLearuWrP9MDH5MBPbIqV92AaeXatLxBI9gBaebbnrfifHhDYfgasaacH8akY=wiFfYdH8Gipec8Eeeu0xXdbba9frFj0=OqFfea0dXdd9vqai=hGuQ8kuc9pgc9s8qqaq=dirpe0xb9q8qiLsFr0=vr0=vr0dc8meaabaqaciaacaGaaeqabaqabeGadaaakeaacuWG4baEgaGaaaaa@2E2E@ are state variable and its rate of change in time; *a, b, c, d *are parameters and *a *> 0. The terms *cx *and *du *indicate respectively the indirect (adaptive/dynamic) effect and direct (feedforward reflex) effects of the input on the output. For a constant-step input, *u *≡ constant for 0 <*t *<*T *where *T *is the end of input, the solution for Eq. 1 under zero initial condition for *x *is:

*y*(*t*) = *du *+ (*cbu*/*a*)(1 - *e*^-*at*^)for 0 <*t *≤ *T *    (2)

This model represents an integrator (Fig. 5A, left panel) if both terms on the right hand side of Eq. 2 have the same sign, or a differentiator (Fig. 5A, right panel) if they have opposite signs. An integrator or differentiator is said to be *inverted *(with negative gain) if the input exerts an opposite direct effect on the output, i.e., *d *< 0. It is called *biphasic *or *monophasic *depending on the presence or absence of a post-stimulus response (for *t *>*T*):

Biphasic: *y*(*t*) = [*y*(*T*) - *y*(0)]*e*^-*a*(*t*-*T*) ^for *t *>*T *    (3a)

Monophasic: *y *= 0 for *t *>*T *    (3b)

where *y*(0), *y*(*T*) are respectively the outputs at the beginning and end of the step input.

From linear systems theory [145], the equivalent transfer function for the model of Eq. 1 is (Fig. 5B):

Y(s)=d(s+a+cb/d)s+a⋅U(s)     (4)
 MathType@MTEF@5@5@+=feaafiart1ev1aaatCvAUfKttLearuWrP9MDH5MBPbIqV92AaeXatLxBI9gBaebbnrfifHhDYfgasaacH8akY=wiFfYdH8Gipec8Eeeu0xXdbba9frFj0=OqFfea0dXdd9vqai=hGuQ8kuc9pgc9s8qqaq=dirpe0xb9q8qiLsFr0=vr0=vr0dc8meaabaqaciaacaGaaeqabaqabeGadaaakeaacqWGzbqwcqGGOaakcqWGZbWCcqGGPaqkcqGH9aqpdaWcaaqaaiabdsgaKjabcIcaOiabdohaZjabgUcaRiabdggaHjabgUcaRiabdogaJjabdkgaIjabc+caViabdsgaKjabcMcaPaqaaiabdohaZjabgUcaRiabdggaHbaacqGHflY1cqWGvbqvcqGGOaakcqWGZbWCcqGGPaqkcaWLjaGaaCzcamaabmGabaGaeGinaqdacaGLOaGaayzkaaaaaa@4C71@

where *s *is the complex frequency and *Y*, *U *are the Laplace transforms of *y*, *u*, respectively.

## APPENDIX II. Nonassociative-learning integrator/differentiator models

From Eq. 2 the response of a primary integrator or differentiator (Fig. 6) to a step input *u*_1 _applied to the primary pathway is given by:

y1(t)=d1u1+(b1c1u1/a1)(1−e−a1t)for 0<t<T     (5)
 MathType@MTEF@5@5@+=feaafiart1ev1aaatCvAUfKttLearuWrP9MDH5MBPbIqV92AaeXatLxBI9gBaebbnrfifHhDYfgasaacH8akY=wiFfYdH8Gipec8Eeeu0xXdbba9frFj0=OqFfea0dXdd9vqai=hGuQ8kuc9pgc9s8qqaq=dirpe0xb9q8qiLsFr0=vr0=vr0dc8meaabaqaciaacaGaaeqabaqabeGadaaakeaafaqabeqacaaabaGaemyEaK3aaSbaaSqaaiabigdaXaqabaGccqGGOaakcqWG0baDcqGGPaqkcqGH9aqpcqWGKbazdaWgaaWcbaGaeGymaedabeaakiabdwha1naaBaaaleaacqaIXaqmaeqaaOGaey4kaSIaeiikaGIaemOyai2aaSbaaSqaaiabigdaXaqabaGccqWGJbWydaWgaaWcbaGaeGymaedabeaakiabdwha1naaBaaaleaacqaIXaqmaeqaaOGaei4la8Iaemyyae2aaSbaaSqaaiabigdaXaqabaGccqGGPaqkcqGGOaakcqaIXaqmcqGHsislcqWGLbqzdaahaaWcbeqaaiabgkHiTiabdggaHnaaBaaameaacqaIXaqmaeqaaSGaemiDaqhaaOGaeiykaKcabaGaeeOzayMaee4Ba8MaeeOCaiNaeeiiaaIaeGimaaJaeyipaWJaemiDaqNaeyipaWJaemivaqfaaiaaxMaacaWLjaWaaeWaceaacqaI1aqnaiaawIcacaGLPaaaaaa@5E32@

where the subscript '1' indicates attribute to the primary pathway. Because of the input-gating effect (Sect. 4.4.1) the primary pathway is silenced once the primary input is off (for *t *>*T*).

A secondary integrator or differentiator is described by the following model equation:

x˙
 MathType@MTEF@5@5@+=feaafiart1ev1aaatCvAUfKttLearuWrP9MDH5MBPbIqV92AaeXatLxBI9gBaebbnrfifHhDYfgasaacH8akY=wiFfYdH8Gipec8Eeeu0xXdbba9frFj0=OqFfea0dXdd9vqai=hGuQ8kuc9pgc9s8qqaq=dirpe0xb9q8qiLsFr0=vr0=vr0dc8meaabaqaciaacaGaaeqabaqabeGadaaakeaacuWG4baEgaGaaaaa@2E2E@_2 _= -*a*_2_*x*_2 _+ *b*_2_*u*_2 _+ *b*_3_*u*_1_

*y*_2 _= *c*_2_*x*_2 _+ *d*_2_*u*_2 _    (6)

where the subscript '2' indicates attribute to the secondary pathway and *b*_3 _represents the influence of the primary stimulus on the secondary pathway. This model represents an integrator or differentiator if *b*_2 _and *b*_3 _have the same or opposite signs, respectively. Assuming a tonic bias input *u*_2 _in the secondary pathway and a step input *u*_1 _in the primary pathway, the response of the above secondary integrator or differentiator is:

y2(t)=y2(0)+(b3c2u1/a2)(1−e−a2t)for 0<t<T     (7a)
 MathType@MTEF@5@5@+=feaafiart1ev1aaatCvAUfKttLearuWrP9MDH5MBPbIqV92AaeXatLxBI9gBaebbnrfifHhDYfgasaacH8akY=wiFfYdH8Gipec8Eeeu0xXdbba9frFj0=OqFfea0dXdd9vqai=hGuQ8kuc9pgc9s8qqaq=dirpe0xb9q8qiLsFr0=vr0=vr0dc8meaabaqaciaacaGaaeqabaqabeGadaaakeaafaqabeqacaaabaGaemyEaK3aaSbaaSqaaiabikdaYaqabaGccqGGOaakcqWG0baDcqGGPaqkcqGH9aqpcqWG5bqEdaWgaaWcbaGaeGOmaidabeaakiabcIcaOiabicdaWiabcMcaPiabgUcaRiabcIcaOiabdkgaInaaBaaaleaacqaIZaWmaeqaaOGaem4yam2aaSbaaSqaaiabikdaYaqabaGccqWG1bqDdaWgaaWcbaGaeGymaedabeaakiabc+caViabdggaHnaaBaaaleaacqaIYaGmaeqaaOGaeiykaKIaeiikaGIaeGymaeJaeyOeI0Iaemyzau2aaWbaaSqabeaacqGHsislcqWGHbqydaWgaaadbaGaeGOmaidabeaaliabdsha0baakiabcMcaPaqaaiabbAgaMjabb+gaVjabbkhaYjabbccaGiabicdaWiabgYda8iabdsha0jabgYda8iabdsfaubaacaWLjaGaaCzcamaabmGabaGaeG4naCJaeeyyaegacaGLOaGaayzkaaaaaa@5FBE@

and y2(t)=y2(0)+[(y2(T)−y2(0)]e−a2(t−T)for t>T     (7b)
 MathType@MTEF@5@5@+=feaafiart1ev1aaatCvAUfKttLearuWrP9MDH5MBPbIqV92AaeXatLxBI9gBaebbnrfifHhDYfgasaacH8akY=wiFfYdH8Gipec8Eeeu0xXdbba9frFj0=OqFfea0dXdd9vqai=hGuQ8kuc9pgc9s8qqaq=dirpe0xb9q8qiLsFr0=vr0=vr0dc8meaabaqaciaacaGaaeqabaqabeGadaaakeaafaqabeqacaaabaGaemyEaK3aaSbaaSqaaiabikdaYaqabaGccqGGOaakcqWG0baDcqGGPaqkcqGH9aqpcqWG5bqEdaWgaaWcbaGaeGOmaidabeaakiabcIcaOiabicdaWiabcMcaPiabgUcaRiabcUfaBjabcIcaOiabdMha5naaBaaaleaacqaIYaGmaeqaaOGaeiikaGIaemivaqLaeiykaKIaeyOeI0IaemyEaK3aaSbaaSqaaiabikdaYaqabaGccqGGOaakcqaIWaamcqGGPaqkcqGGDbqxcqWGLbqzdaahaaWcbeqaaiabgkHiTiabdggaHnaaBaaameaacqaIYaGmaeqaaSGaeiikaGIaemiDaqNaeyOeI0IaemivaqLaeiykaKcaaaGcbaGaeeOzayMaee4Ba8MaeeOCaiNaeeiiaaIaemiDaqNaeyOpa4JaemivaqfaaiaaxMaacaWLjaWaaeWaceaacqaI3aWncqqGIbGyaiaawIcacaGLPaaaaaa@608F@

Finally, the resultant response of the dual-process integrator and/or differentiator is the sum of *y*_1 _and *y*_2_:

*y *= *y*_1 _+ *y*_2 _    (8)

## Authors' contributions

CSP conceived the theory, reviewed and integrated the literature and wrote the manuscript. DLY performed the mathematical modeling and prepared the illustrations. Both read and approved the final manuscript.
